# Factors associated with compliance of prenatal iron folate supplementation among women in Mecha district, Western Amhara: a cross-sectional study

**DOI:** 10.11604/pamj.2015.20.43.4894

**Published:** 2015-01-15

**Authors:** Bekele Taye, Gedefaw Abeje, Alemetsehaye Mekonen

**Affiliations:** 1 Bahir Dar University College of Medicine and Health Sciences, Bahirdar, Ethiopia

**Keywords:** Compliance, iron folate supplementation, mecha district, amhara region

## Abstract

**Introduction:**

Iron and folate supplementation can effectively control and prevent anaemia in pregnancy. In Ethiopia, all pregnant women are prescribed iron folate during their ANC visit. However, limited adherence is thought to be a major reason for the low effectiveness of iron supplementation programs. Therefore this study was done to investigate factors associated with compliance of prenatal iron folate supplementation among women who gave birth in the last 12 months before the survey in Mecha district.

**Methods:**

Community based cross sectional study design was employed in Mecha district from June 25 - July 15/2013. A sample of 634 women who gave birth 12 months before the survey was included in the study. Study participants were selected by systematic random sampling technique after allocating the total sample to each kebele proportionally. Data were collected using a pre-tested structured Amharic questionnaire. Collected data were edited, coded and entered to Epi info version 3.1 and exported to‘ SPSS version 16. Bivariate and multivariable analysis was computed.

**Results:**

A total of 628 women who gave birth twelve months before the survey were enrolled. In this study only 20.4% of participants were compliant with iron foliate supplementation. In multivariable analysis, age of the mother, educational status of the mother, knowledge of anaemia and iron folate tablets, and history of anaemia during pregnancy were significantly associated with compliance to iron folate supplementation (P < .05). Belief that too many tablets would harm the baby and fear of side effects were the major reasons given for noncompliance.

**Conclusion:**

Compliance to iron folate supplementation is very low in the study area. Increasing female education and increasing knowledge of women about anaemia and iron folate tablets are recommended to increase compliance to iron folate supplementation.

## Introduction

Anaemia is a global public health problem affecting both developing and developed countries with major consequences for human health [1]. Around 2 billion people, accounting to over 30% of the world's population are anaemic, mainly due to iron deficiency [2]. Iron deficiency is the most prevalent and also the most neglected nutrient deficiency in the world, particularly among pregnant women and children, especially in developing countries [3]. Foliate deficiency has been also associated with anaemia and other adverse outcomes like neural tube defects in pregnancy. Folic acid supplementation and food fortification are currently the feasible strategies recommended to prevent such outcomes [4]. A study done in Ethiopia on prevalence of iron deficiency anaemia among pregnant and lactating women showed that 18.7% of women had iron deficiency anaemia [5]. According to the 2005 and 2011 Ethiopian Demographic and health surveys, the prevalence of anaemia among pregnant women was 30.6% and 27% respectively [6, 7]. Experts suggest that 1000 mg of iron are needed for the mother and fetus during pregnancy, a requirement that is practically impossible to meet with most diets in developing countries [8]. Because many women enter pregnancy with little iron store, the recommendation of daily oral iron supplements has been a routine practice in primary health care units [9]. Many developing countries are now implementing iron supplementation programs, but only a few countries have reported significant improvement in anaemia control and prevention [10]. Although clinical trials have repeatedly shown the efficacy of supplementation, most if not all community-based programmes have not been shown to be effective, i.e. they have not decreased the incidence and prevalence of anaemia when deployed in the field.

Studies conducted in South-East Asia, Latin America and in few African countries have shown that one of the main reasons why these programmes have been less effective than anticipated is low compliance of women with taking daily iron supplements. Low compliance has been associated with a number of factors, including: gastrointestinal side effects that can occur with taking iron, inadequate supply of tablets (including limited resources to purchase tablets), inadequate counselling of patients by healthcare providers concerning the utility of tablets and possible transient side-effects, poor utilisation of prenatal health-care services, lack of knowledge and/or patient fears about the tablets and community beliefs, attitudes and practices that affect women's perception regarding tablet use [11]. In Ethiopia, iron/foliate supplementation is the main strategy for anaemia control and prevention. However, adherence rate remains very low. A survey done in Ethiopia revealed that only 16.1% of pregnant women took the iron/foliate supplements for 90 or more days [12]. Even though adherence is a major problem in iron-supplementation programs, limited researches have been done on this topic. Moreover, no studies done on compliance of iron foliate supplementations in Amhara region. Therefore this study was done to determine compliance rate and to identify factors associated with compliance with iron/folate supplementation among women in Mecha district.

## Methods

### Study setting

A community based cross sectional study was employed using quantitative method of data collection in Mecha district, Western Amhara from June to July 2013. The district is one of the 15 districts of West Gojjam Zone in Amhara regional state. It is located 35kms South West of Bahir Dar on the main road from Bahir Dar to Addis Ababa. The total population of the district was about 323,950. Of the total population about 161003(49.7%) were males and 162947(50.3%) were females. The district had 40 rural and 4 urban Kebeles (the smallest administrative unit). The district has a total of 10 health centre and 40 health posts. There are five NGOs working on maternal and new born health in the district [13].

### Sampling

The source population of the study was all women who gave birth twelve months before the survey and who were residents of Mecha district. Sample size was calculated using a single population proportion formula 50% compliance to iron folate supplementation, of p (0.5), 5% margin of error (d) and 95% (za/2 = 1.96) confidence level. After considering design effect of 1.5 and non-response rate of 10%, the final sample size was 634.

### Sampling technique

A multi stage stratified sampling technique was used with the strata of kebeles. A total of 9 kebeles, one from urban and eight from rural were randomly selected and included in the study. The total sample size was allocated proportionally to each kebele based on the number of deliveries in each kebele 12 months before the survey. Then, respondents were selected by systematic random sampling from health extension workers registration book. Data were collected using structured Amharic (Ethiopian national language) questionnaire through interview by trained nurses supervised by health officers. The questionnaire had four sections: socio-demographic characteristics of participants, reproductive history, obstetrical and medical complications during pregnancy, and knowledge of anemia and iron foliate supplementation. In this study, a woman was considered as compliant to iron/folate supplementation if she had taken ≤90 iron folate tablets on daily basis during her pregnancy period [12, 14, 15]. Knowledge of anemia was measured by a questionnaire composed of 10 questions. The questions ask about causes, symptoms, treatment and prevention of anemia. The correct answer was given a score of one and the incorrect was given a score of 0. The woman's knowledge to anaemia was classified as high, medium and low if she could give correct answer to 8 -10, 6-7 and less than 6 questions respectively [16]. Women's knowledge about iron folate supplementation was assessed by 7 questions. A woman who answered more than 80% of the questions correctly were classified as women with high knowledge about iron folate supplementation, those who answered 60%-70% of the questions correctly were considered women with moderate knowledge and those who answered less than 60% of the questions correctly were classified as less knowledgeable about iron folate supplementation. One day training regarding the objectives of the study and ways of administering the questionnaire was given to the data collectors and supervisors by the investigator. The prepared questionnaire was pre-tested prior to the actual data collection on 30 participants that were not included in the main survey. The data was edited, coded, and entered using Epi data version 3.1 and exported to SPSS version 16 for analysis.

### Data analysis

Bivarate and multivariable analyses were computed to test whether there was association between compliance of iron folate supplementation and selected independent variables.

### Ethical consideration

Ethical clearance was obtained from ethical review committee of Bahir Dar University College of Medicine and health sciences. Letter of permission was obtained from the Amhara regional state health bureau and Mecha district health office. The confidentiality of information was maintained by excluding personal identifiers and by conducting the interview privately. Data were collected after securing informed consent from every respondent.

## Results

### Socio demographic characteristics

From a total of 634 women included in the study, 628 respond to the questioners, giving a response rate of 99%. The mean age of women was 27 with standard deviation of ±5.27 years old. More than half of the women (57.2%) were aged 25-34 years. Nearly all participants (98.7%) were Orthodox Christian followers. From the total women who responded to the questionnaire, 92.5% were rural residents. Three hundred seventy five (59.7%) of participants were unable read and write. Most of the study participants (96.5%) were married. About half (53.3%) of women earned? 500 Ethiopian birr monthly. Regarding their occupation, five hundred eight (80.9%) women were farmers while only 4 (0.6%) were government employees (Table 1).

**Table 1:** Socio-demographic characteristics of women who gave birth in last 12 months before the study in Mecha district, Western Amhara, July, 2013.(N = 628)

Variables	Category	Frequency	percentage
Age	15-24 years	208	33.1
	25-34 years	359	57.2
	35-49 years	61	9.7
Religion	Orthodox	620	98.7
	Muslim	8	1.3
Residence	Rural	584	93
	Urban	44	7
Educational level	Unable to read and write	375	59.7
	Able to read and write	165	26.3
	Elementary completed	68	10.8
	Secondary completed	14	2.2
	College and above	6	1.0
Marital status	Married	606	96.5
	Others*	22	3.5
Monthly income in Et. Birr	≤500	335	53.3
	500-1000	263	41.9
	≥1000	30	4.8
Current occupation	Farmer	508	80.9
	Housewife	35	5.6
	Merchant	71	11.3
	Government employee	4	.60
	Others**	10	1.6

* others **(**Widowed, Divorced and Single)

** Others (daily labourer, private employer, student)

### Obstetric and medical history of mothers

From the study participants, 74% were multi gravid and 26% were primi gravid. The study found that among multi gravid women, 93.5% received ANC follow up for their previous birth. Among women who had ANC follow up, 5.5% had received only one ANC for the current pregnancy. The majority of the women (93.3%) attended ANC follow up in health centre. Regarding history of medical problem during current pregnancy, about 24.2% of women reported history of anaemia, 6.5% reported history of malaria attack, 2.5% reported history of hypertension and 6.7% reported history of other pregnancy complication during the recent pregnancy (Table 2).

**Table 2:** Obstetrics and medical history of women who gave birth 12 months beforethe study in Mecha district, Western Amhara, July, 2013

Variable	Category	Frequency	Percentage
ANC follow up for Previous birth	Yes	435	93.5
	No	30	6.5
	Total	465	100
No of ANC visit in current birth	Only 1	35	5.5
	Only 2	163	26.0
	Only 3	289	46
	Only 4^+^	141	22.5
	Total	628	100
Place of ANC care receive for current pregnancy	Health centre	586	93.3
	Health post	42	6.7
	Total	628	100
History of anaemia during current pregnancy	Yes	152	24.2
	No	476	75.8
	Total	628	100
History of malaria attack during current pregnancy	Yes	41	6.5
	No	587	93.5
	Total	628	100
History of hypertension during current pregnancy	Yes	16	2.5
	No	612	97.5
	Total	628	100
History of any complication during current pregnancy	Yes	42	6.7
	No	586	93.3

### Knowledge of anaemia and iron folate supplementation tablets

In this study, only 27.4% of women had higher level of knowledge for anaemia. This study also showed that only 24.8% of women had higher knowledge about iron folate tablets.

### Compliance to Iron/Foliate Supplementation

In this study, only 128 (20.4%) women were compliant with iron/folate supplementation (took iron folate tablets for 90 days during entire pregnancy). The total tablets taken range from 3 tablets to 120 tablets with a median of 65 tablets (SD = 22.63). The majority of women (71.8%) reported that they were taking the supplement on daily basis and the other 177(28.2%) used to miss 1 or more tablets per week.

### Reasons for compliance or non compliance to Iron/Folate Supplementation

In this study, the reasons for compliance (to continue taking iron tablets) were clinician instruction, adequate explanation about the tablets by providers (65.6%) and fear of illness if missed (57%). About 44.5% comply because they thought that the tablets would'increase their blood. Other reasons mentioned for compliance were family support to take the tablet (12.5%) and getting the tablet free of charge. The most commonly cited reasons for non compliance were fear that too many tablets would harm the mother and/or her baby (58%), side effects (54.4%) and fear that babies will become bigger (Table 3).

**Table 3:** Reasons for non compliance of iron folate supplementation among women who gave birth in last 12 months before the study in Mecha district, Western Amhara, July, 2013(N = 500)

Reasons (multiple response possible)	Frequency	Percentage
Fear that too many tablets would harm mother and/or her baby	290	58
Side effects	272	54.4
Fear that baby will become bigger	142	28.4
Family influence	77	15.4
Similar pill (colour, taste)	60	12
Forgetfulness	46	9.2
Negligence(poor attention to importance of IRF)	34	6.8
No need to take after the symptom relieved	31	6.2
Lost the prescription/tablets and were afraid to ask for another	19	3.8
Others*	16	3.2

### Factors associated with compliance to Iron/Folate Supplements

BIivariate and multivariable logistic regression was done to identify factors associated with compliance of iron foliate supplementation. First all factors were analyzed by bivariate analysis. Only seven factors showed significant association with compliance. Then variables with P-value ≤0.2 in the bivariate analysis were entered into multi variable logistic regression analysis. Among these, five predictors were found significantly associated with iron/folate supplementation. The multivariable analysis indicated that women aged 35-49 years old were 3.41 times more likely to comply compared to women aged 15-24 years old (AOR: 3.42, 95%CI: 1.44-8.16). In this study, literate women were 4.49 times more likely to be compliant compared to women who were unable to read and write (AOR: 4.49, 95%CI: 2.61-7.76). Women who reported history of anaemia during this pregnancy were almost 6 times more likely to be compliant compared to those women who did not report history of anaemia. (AOR: 5.83, 95%CI: 3.41-9.96). Similarly, women who had higher knowledge of anaemia were almost four times more likely to comply to iron foliate supplementation compared to women who had low knowledge (AOR: 3.64, 95%CI: 1.78-7.39). Those women who had higher knowledge of iron foliate tablets were 5.25 times more likely to be compliant to iron foliate supplementation compared to those women who had low knowledge (AOR:5.25,95%CI:2.72-10.15) (Table 4).

**Table 4:** Factors associated with iron folate supplementation compliance among women who gave birth in last 12 months before the study in Mecha district, Western Amhara, July, 2013

Variables	Compliance =128)	Non-compliance N = 500)	COR(95%CI)	AOR(95%CI)
Age of women in years				
15-24	41	167	1.00	1.00
25-34	64	295	0.88(0.57-1.37)	1.14(0.64-2,02)
35-49	23	38	2.47(1.33-4.58)	3.42(1.42-8.16)^**^
History of anaemia during this pregnancy				
Yes	78	74	8.98(5.83-13.84)	5.83(3.41-9.96)**
No	50	426	1.00	1.00
History of malaria attack during this pregnancy				
Yes	16	25	2.71(1.40-5.25)	1.12(0.45-2.81)
No	112	475	1.00	1.00
Knowledge of anaemia				
Higher	88	84	8.77(3.59-30.56)	3.64(1.78-7.39)**
Moderate	24	282	0.71(0.36-1.38)	0.54(0.25-1.14)
low	16	134	1.00	1.00
Knowledge of iron foliate tablets				
Higher	75	81	10.35(6.05-17.72)	5.25(2.72-10.15)**
Moderate	31	173	2.00(1.12-3.58)	1.46(0.74-2.88)
low	22	246	1.00	1.00

## Discussion

Compliance with iron supplementation plays a major role in the prevention and treatment of iron deficiency anaemia particularly among pregnant women whose iron requirement starts at the second trimester and progresses until the third trimester. Thus, this study attempted to investigate factors associated with compliance of prenatal Iron folate supplementations. The result revealed that 20.4% women were compliant (took iron folate tablets for 90 or more days during entire pregnancy) to the supplementation, which is higher compared with the study done in four regions of Ethiopia which was 16% [12]. The probable reason may be the difference in geographic locations and time gap between studies, and inaccessibility of health institutions. However, it is much lower than WHO's and Ethiopia's National Guidelines for Control and Prevention of Micronutrient Deficiencies [17, 18]. The compliance rate among women in this study was still lower compared to studies done in Asia, Senegal and India [14, 15, 19]. This difference may be due to differences in awareness of pregnant women about iron folate supplementation and educational status. In addition, low attention to compliance issue due to lack of information regarding the severity and magnitude of the problem and poor follow up of the program in this study area may be the other reason for this difference. For instance, no indicators that are used to assess compliance/ adherence of iron foliate supplementation in the Ethiopian health service reporting system. In this study, it was observed that 58% of mothers stopped taking the supplementation because they thought that too many tablets would harm her and/or her baby. This may indicate lack of awareness about iron/folate tablets among pregnant women and poor counselling services about the importance of supplementation. Fifty four percent of women stopped taking the supplementation due to fear of side effects which is higher than studies done in India, Senegal and Vientiane which were 29.3%, 26.8% and 18.38% respectively [14, 15, 20]. This difference may be due to the fact that the present study considers multiple responses but the previous studies allowed only on reason. In addition to this, provider counselling about possible side effects of the supplement may be the cause for the difference. In this study, counsellors were midwifes/nurses and health extension workers contrary to the previous studies in which supplementation were provided only by midwifes/nurse [14, 15, 20].

Beliefs about health and treatment may have also interfered with iron compliance. In this study, 28.45% of women believed that continuous taking of iron folate supplementation leads to over-weight babies. The finding of this study is supported by a study done in Thailand in which some women decided not to take supplements because they thought that iron causes the babies to be over-weight [21]. On the other hand, this finding was higher compared to study done in Vientiane which was 13% [20]. This could be due to differences in educational status of pregnant women, dissemination of information and awareness on the benefits of the supplement. This study also found that reasons of non compliance due to forgetfulness accounts for 9.2% which is consistent with a study done in India which was 9.68% [15], but it was lower than a study in Senegal which was 17.2% [14]. Another study done in Ethiopia in four regions indicated that the major reasons for non-adherence were forgetfulness, fear/experience of side effect, fear of drug intake during pregnancy and lack of awareness on the benefits of the supplement [12]. According to this study, major reasons for compliance were clinicians’ instructions and order to take the tablets (65.6%), taking whatever prescribed (57%), knowing that the tablets would'increase their blood (44.5%), and family support (12.5%) which is consistent with a study done in Senegal [14]. In addition, a study conducted in Burma indicated that providing appropriate language services, providing child care facilities and bringing treatment programs to the work-place increases compliance to supplements [22].

Compliance may be influenced by a number of factors. One factor shown to have a significant association with the compliance was the participants’ age. Women aged 35-49 years old were three times more likely to be compliant to iron foliate supplementation than women with younger ages(15-24 years) (AOR: 3.42, 95%CI: 1.44-8.16). The reason for this is that older women may be more concerned about their health and pregnancy outcome, get necessary support and cooperation from their family members and had better experience in prevention and treatment of iron deficiency anaemia. This finding was in line with a study in India that elderly and middle women were slightly more compliant than younger women [15]. Education enables patients to appreciate the benefits that can be gained from complying with a medical regimen and can influence the overall health seeking behaviour of women. In this study, literate women were four times more likely to comply compared to women who are unable to read and write (AOR: 4.49, 95%CI: 2.61-7.76). The reason is that educated women have better knowledge about the iron deficiency anaemia and therapy, better knowledge of the benefits of supplements and increased concern about pregnancy outcome. This finding is supported by a study done in India [15]. History of anaemia had also shown significant association with the participants’ compliance to iron folate; women who reported history of anaemia during pregnancy were five times more likely to be compliant compared to those who did not report (AOR: 5.83, 95%CI: 3.41-9.96). This could be due to relief from symptoms, fear of further complications and provider may give more emphasis for anaemic clients on counselling compared to non-anaemic clients. in this study, women who had higher knowledge of anaemia were three times more likely to be compliant compared to those who had low knowledge (AOR: 3.64, 95%CI: 1.78-7.39). Similar results were reported in Asia and Vientiane in which women with good knowledge of anaemia were more likely to be more compliant [18, 20]. Women who had higher knowledge of iron foliate tablets were 5.4 times more likely to be compliant compared to those who had low knowledge (AOR: 5.25, 95%CI: 2.72-10.15). The reason could be knowledge helps women to have a good perception of benefits of taking iron tablets. Some limitations need to be considered when interpreting the results of this research. First, reports by the women may under/overestimate compliance rate since the data collected were by self report. A second limitation is the fact that health conditions were based on self-reports, thus may be over or underestimated.

## Conclusion

Compliance of prenatal iron folate supplementation remains very low in the district and does not meet WHO recommendations despite the usefulness of this health program to combat iron deficiency anaemia. Age of the mother, educational status, knowledge of anaemia and iron folate tablets, and history of anaemia during pregnancy were found to be significantly associated with compliance of prenatal iron foliate supplementation. In addition, fear that too many tables would harm the mother and/or the baby, fear that the baby will become bigger and side effects were the most commonly mentioned reasons for failure to comply with iron folate supplementation. Therefore, pregnant woman should receive adequate information from health providers about iron folate supplementation. They should be aware of the benefits and importance of taking the supplementation. It is also important to provide information for women about anaemia and iron folate tablets. More thorough and in-depth studies are recommended for designing and refining iron supplementation program strategies and messages for communication materials.
